# Surface analysis and depth profiling using time-of-flight elastic recoil detection analysis with argon sputtering

**DOI:** 10.1038/s41598-018-28726-x

**Published:** 2018-07-10

**Authors:** Zdravko Siketić, Iva Bogdanović Radović, Ivan Sudić, Milko Jakšić

**Affiliations:** Laboratory for Ion Beam Interactions, Department of Experimental Physics, Ruđer Bošković Institute, Bijenička cesta 54, 10000 Zagreb, Croatia

## Abstract

The recent development of new advanced materials demands extensive effort in developing new analytical techniques that can provide insight into material composition at the nanoscale, particularly at surfaces and interfaces, which is important for both fabrication and material performance. Here, we present a proof of principle for a new setup used for thin-film characterisation and depth profiling based on a combination of time-of-flight elastic recoil detection analysis (TOF-ERDA) and Ar sputtering. A quantitative depth profiling with a best achievable surface depth resolution of ~2 nm can be realised for the entire layer, which is important for the precise determination of thickness and composition of samples that are several tenths of a nanometre thick. The performance of TOF-ERDA with Ar sputtering was demonstrated using 15 nm Cu evaporated onto a Si substrate. The advantages and limits of the method are discussed in detail.

## Introduction

Semiconductor devices, sensors, biomaterials, high-density magnetic storage media, thin films and coatings are all currently based on ultra-thin layers and multilayer systems, which are often thinner than 30 nm. Therefore, advanced analytical techniques that can provide information for the composition and depth profiles of elements contained in such layers at the nanoscale level must be developed^1–4^.

The most common analytical techniques used to characterise materials as thin as 10 nm are secondary ion mass spectrometry (SIMS), X-ray photoelectron spectroscopy (XPS) and Auger-electron spectroscopy (AES)^5–9^. One of the promising techniques for surface analysis is time-of-flight elastic recoil detection analysis (TOF-ERDA), which is a well-established ion beam analysis (IBA) technique and a rare technique for the quantitative depth profiling of all elements (including hydrogen) in the first several hundred nm of a sample^10–16^. Heavy ions (such as Cl, I, and Au) with energies of a few tens of MeV are used to recoil atoms from the target. By measuring the energy and time of flight of coincidentally recoiled atoms, all elements can be separated by energy and mass. Then, the time/energy spectra are converted into depth profiles using the known relation for the energy loss per unit of length of ions in the sample (stopping power) and Rutherford cross-section data. More information on TOF-ERDA can be found elsewhere^17^.

The main attributes of TOF-ERDA are the good mass separation of recoiled atoms, which is better than 1 for M < 40 in our case, and an excellent surface depth resolution (as good as 2 nm for low incident angles)^18–20^. Optimisation of each TOF-ERDA measurement can be realised by carefully selecting the scattering geometry together with the incidence ion mass and energy^21^. The optimisation strongly depends on the type of the analysed target (heavy or light matrix) as well as on the information one requires to be extracted from the measurement (elemental depth profiles or total number of atoms). The best possible surface depth resolution in TOF-ERDA can be achieved by the use of a heavy primary ion in combination with a small incidence angle (angle between the ion and sample surface)^21^. However, despite the excellent surface depth resolution, this combination will cause fast deterioration of the depth resolution with the sample depth due to the multiple and dual scattering processes of both incident ion and recoiled atoms^17,20^. To avoid this deterioration, lighter ions with a larger incidence angle can be used; however, this leads to a worsening of the surface depth resolution.

To maintain the best possible surface depth resolution through the entire analysing depth, we have combined TOF-ERDA measurements with sample sputtering using an Ar sputtering gun, which is similar to the process utilised during depth profiling using other techniques (SIMS, XPS and AES). Thus, the Ar sputtering removes a part of the analysed sample, which enables the area appearing at the top surface to be measured with the best achievable surface resolution and reduces the potential effects that deteriorate the depth resolution below the surface such as multiple or plural scattering. Thus, in this setup, TOF-ERDA is used to measure the surface elemental composition only, whereas argon sputtering is applied to extract the depth information.

Compared with other depth-profiling techniques (SIMS, XPS, and AES), TOF-ERDA is fully quantitative; the calculation of elemental concentrations is straightforward and not matrix-dependent. The obtained surface depth resolution in the range of 2 nm is notably similar for SIMS, XPS, and AES^20,22^. In addition, during each subsequent argon sputtering, the surface composition and remaining film thickness are measured, which can inform us about the total amount of sputtered material. Thus, the combination of TOF-ERDA and Ar sputtering does not need to know a sputtering yield in advance to extract the depth information; in addition, the sputtering yield for each material can be directly measured. The sensitivity of the TOF-ERDA is 0.003-0.1 at.%, which is similar to the sensitivity obtained with XPS (0.01 at.%) and AES (0.1 at.%) but much worse than the SIMS sensitivity (10^−3^-100 ppm)^20,22,23^. Since TOF-ERDA is routinely carried out using broad ion beams with a diameter of a few mm, the present setup is not used for imaging. However, because there exist heavy-ion high-energy microprobes with lateral resolutions of 5 µm and below, there is a possibility of developing a system based on an ion microprobe in the future^24–26^.

In the present paper, a new setup for TOF-ERDA with Ar sputtering is studied. Using an Ar beam, depth profiling with the best achievable surface depth resolution can be carried out through the entire sample depth. To test the performance of TOF-ERDA with Ar sputtering, a sample consisting of 15 nm Cu evaporated on Si was used. The advantages and limits of the method are discussed in detail.

## Experimental

To achieve a surface depth resolution of a few nm for TOF-ERDA measurements, a 23-MeV ^127^I^6+^ beam with a 2.5° incidence angle was selected. Recoiled ions from the sample were detected by a TOF-ERDA spectrometer placed at 37.5° with respect to the incident beam direction.

For the Ar sputtering, a Prevac-IS 40C1 gun with an IS40-PS power supply was used. The energy of the argon beam was E(Ar^+^) = 1 keV; the incidence angle towards the sample surface was θ_in._ = 45°; the current density was ≈6 µA/cm^2^; and the Gaussian beam profile had a full width at half maximum (FWHM) of ≈8 mm (all specifications were obtained from the manufacturer). To ensure a homogenous lateral sputtering, the sample was scanned during the argon beam irradiation. The selected beam spot size for the incident iodine ions was much smaller than the sputtered area to avoid potentially inhomogeneous sputtering near the edges.

The new setup (TOF-ERDA + Ar sputtering) was tested on ≈15 nm of Cu evaporated onto a Si substrate with the native oxide on the top (the copper thin film was evaporated using a Leybold UNIVEX 300 evaporator).

Two different energies for the Ar ions were studied (1 and 5 keV) to compare the depth distribution of the recoiled copper atoms. According to SRIM2013^27^, in the case of the 5-keV Ar beam, the FWHM of the recoiled-copper-atom distribution was ≈3 nm, which is larger than the surface depth resolution of TOF-ERDA (≈2 nm). Therefore, a 1-keV Ar beam was selected with an approximately 1.5-nm-FWHM recoil distribution for Cu atoms to reduce as much as possible the ion beam mixing effect on the depth resolution.

The depth profiling of the Cu layer was carried out first using a TOF-ERDA measurement of the sample surface composition followed by argon sputtering (sputtering time of ~10 min or more) for obtaining depth information. Each TOF-ERDA spectrum was analysed using the simulation code Potku and SRIM2013 stopping power data^27,28^ (the depth scale is in units of 10^15^ atoms/cm^2^)^17^.

## Results and Discussion

As mentioned in the previous paragraph, the calculated depth profiles are given in units of atomic concentration vs. depth in 10^15^ atoms/cm^2^. For the depth conversion to nanometres, where applicable, the density of copper (ρ = 8.951 g/cm^3^) was used.

**Figure 1:**
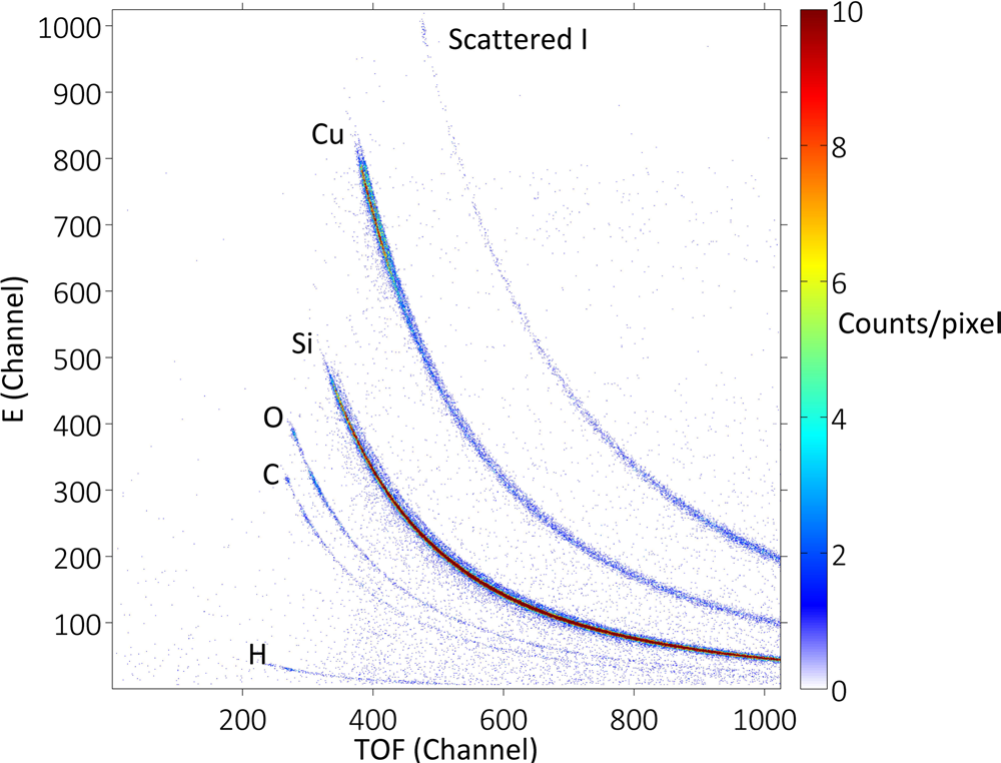
TOF-ERDA coincidence spectra for ≈15 nm Cu/Si.

The TOF-ERDA coincidence spectra for the virgin (non-sputtered) test sample (≈15 nm Cu/Si) are shown in Fig. 1.

**Figure 2:**
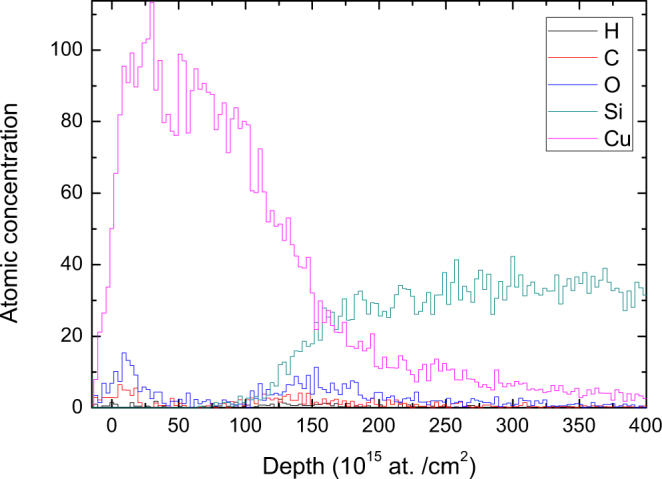
Depth profile for ≈15 nm Cu on a Si substrate from a TOF-ERDA measurement of a virgin (non-sputtered) sample.

The analysed film evidently consists of copper, including hydrogen, carbon and oxygen contamination through the entire evaporated layer. The depth profile, which is related to Fig. 1, calculated using the “slab analysis” software Potku^28^, is shown in Fig. 2. Potku calculates absolute concentrations by integrating the depth profiles over user-defined ranges, taking into account only the cross section and stopping power data. The whole depth profile is scaled to 100% near the sample surface to avoid effects such as energy straggling and multiple scattering effects, which Potku does not take into account.

Oxygen and carbon contaminants are mainly located at the sample surface and interface between the thin film and Si substrate.

Due to multiple scattering and energy straggling, the depth resolution for Cu at the interface is worse than the depth resolution at the surface. By fitting the “error function” for both sides of the layer, the calculated FWHM for the depth resolution is (19 ± 5)*10^15^ at./cm^2^ or (2.2 ± 0.5) nm at the surface and (83 ± 10)*10^15^ at./cm^2^ or (10 ± 1) nm at the interface. The thickness of the Cu film was 130*10^15^ at./cm^2^, which corresponds to 15.3 nm.

The obtained results show that depth resolution is 4.5 times worse at the interface, which is only 15.3 nm away from the surface. This result clearly illustrates how fast the depth resolution deteriorates with sample thickness if the TOF-ERDA measurement is carried out using a heavy ion in glancing incidence geometry. On the other hand, an excellent surface depth resolution of 2.2 nm obtained by the TOF-ERDA measurement is confirmed.

Another multiple-scattering effect is based on the fact that the Si concentration is much below the expected value (well below 100%) and mixed with the multiple-scattering tail of Cu. This part of the sample is the substrate bulk, and only Si should be present in the bulk with a concentration of 100%.

After measurement of the depth profile for the virgin sample using only TOF-ERDA (starting point), subsequent Ar sputtering and TOF-ERDA measurements were performed. For each TOF-ERDA measurement, the surface composition, depth resolution and film thickness were calculated. From the remaining film thickness, the amount of sputtered material is calculated to provide one with depth information.

**Figure 3:**
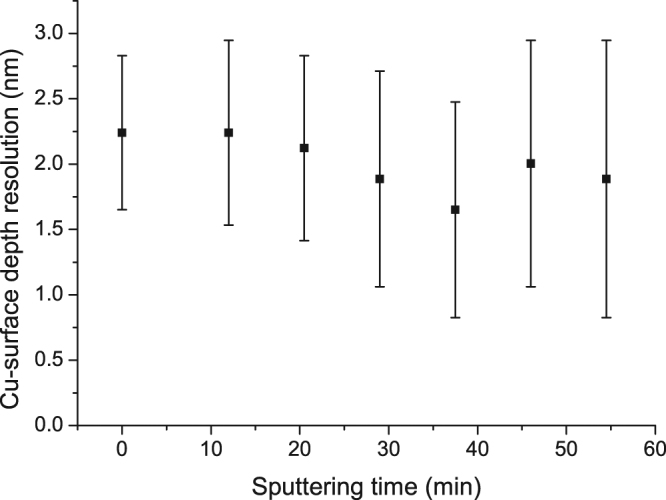
TOF-ERDA surface depth resolution of copper for the first 6 sputtering cycles.

To check whether the proposed technique will affect the superior surface resolution of TOF-ERDA, we measured the surface resolution for several subsequent sputtering periods, as shown in Fig. 3.

The TOF-ERDA surface depth resolution does not change during an Ar sputtering time of less than 60 min.

**Figure 4:**
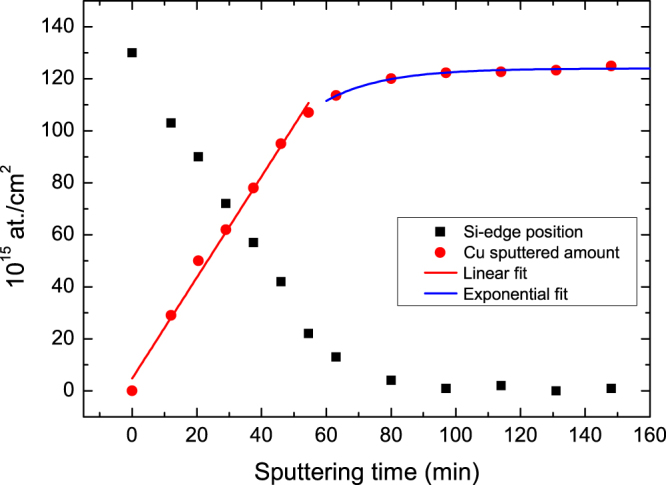
Amount of sputtered Cu (in 10^15^ at./cm^2^) and position of the silicon edge (in 10^15^ at./cm^2^) as a function of the Ar sputtering time. Linear and exponential fit of the sputtering rate of copper are also shown.

The amount of sputtered copper (in 10^15^ at./cm^2^) and position of the silicon edge (in 10^15^ at./cm^2^) with sputtering time is shown in Fig. 4.

As shown, the Si edge is approaching the surface for an increasing amount of sputtered Cu.

In the region of 0–54.5 min, the Cu sputtering is linear with sputtering time. For this sputtering range, the sputtering rate of copper is calculated to be (0.32 ± 0.01)*10^15^ at.min^−1^µA^−1^; if the copper density is used, (0.038 ± 0.001) nm min^−1^µA^−1^cm^2^, the Ar current density is ≈6 µA/cm^2^, as provided by the manufacturer. The sputter yield for copper (0.32 ± 0.01)*10^15^ at.min^−1^µA^−1^ (or ~0.85 Cu atoms/Ar ion) is ~3 times lower than that reported by Seah *et al*.^29^. It may be speculated that uncertainty in the given Ar beam current density (≈6 µA/cm^2^), which we were not able to measure during the sputtering time, can cause this difference.

The exponential behaviour of the sputtered amount of Cu with sputtering time for t_sputt._ >60 min can be explained as follows: when the region near the interface is sputtered, a certain amount of Cu atoms is sputtered away; simultaneously, part of the Cu atoms is recoiled by Ar in the Si substrate (ion beam mixing). The amount of “implanted” atoms at time t is proportional to the total amount of atoms in the thin layer at the interface. This behaviour is a simple exponential decay law, and a similar description was used to describe the ion beam mixing in high-resolution AES depth profiling^6,9,30^.

**Figure 5:**
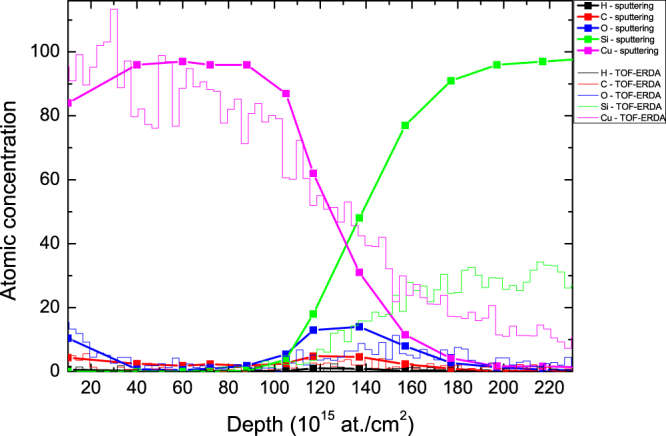
TOF ERDA promoted by argon sputtering (lines + symbols) and TOF-ERDA (lines) depth profile for a 15-nm-thick Cu layer evaporated onto the Si substrate.

The final result of TOF-ERDA promoted by Ar sputtering on the thin Cu layer on the Si substrate is shown in Fig. 5. Lines + symbols present the depth profile of the sputtered sample, where the surface composition was measured using TOF-ERDA (averaging over depth, within depth resolution), for each depth obtained by Ar sputtering. In addition, the depth profile of the virgin sample (non-sputtered), which is only measured by TOF-ERDA and analysed by Potku, is shown for comparison (lines).

The data in Fig. 5 show that in case of TOF-ERDA promoted by Ar sputtering, the depth profile is measured with surface depth resolution, which is the best depth resolution through the entire analysing depth, regardless of the film thickness (see Fig. 3). The depth resolution at the interface is in the range of ~50*10^15^ at./cm^2^ (or ~5.5 nm), which is better than that for TOF-ERDA only (~10 nm) but is worse than the surface depth resolution (~2 nm). Two effects can be responsible for this observation: the first is recoil implantation of Cu in Si (maximum penetration range of ~3 nm), and the second is the estimated surface roughness coming from the argon sputtering after a long sputtering time (~4 nm). Another important advantage of using the combined techniques is that the Cu depth profile is not decreased with depth as it is in the case when solely TOF-ERDA is used. This artificial decrease is caused by the multiple scattering of Cu ions that emerge from the deeper part of the sample and that are deflected out of the spectrometer solid angle. The same scattering effects leads to a Si concentration well below 100%, which is also corrected by combining TOF-ERDA and the sputtering measurements. The concentration depth profile for the oxygen at the interface is more accurate while multiple scattering effects are completely excluded. The oxygen interface position (~125*10^15^ at./cm^2^) is shifted more towards the surface than the TOF-ERDA depth profile is (~140*10^15^ at./cm^2^) for the non-sputtered sample. This discrepancy results from the uncertainty in the SRIM2013 stopping power database for heavy ions^27^.

It must be mentioned that due to the glancing incidence geometry (2.5° with respect to the sample surface), the sample to be analysed must be extremely flat, because any surface roughness in the range of ~5 nm can lead to rapid deterioration of the depth resolution. Additionally, due to the need for reasonable statistics in each repeated TOF-ERDA measurement, a cumulative ion beam dose can lead to sample decomposition. This effect could be a problem, and the limiting factor for the proposed technique, when very thin samples, with a low concentration of light elements, are analysed. However, selection of heavy primary ions is here beneficial. The resulting “damage cross section” for heavy ions (stopping power divided by scattering cross section) is smaller, with possible ion beam damage for the given measuring time/statistics reduced.

## Conclusions

We have demonstrated that TOF-ERDA with argon sputtering can be a notably useful technique for measuring the concentrations and depth profiles of all elements in the surface layers and interfaces of thin films (~15 nm). The elements can be more accurately profiled through the entire depth using the best possible depth resolution – surface depth resolution. Additionally, the sputtering yield for different elements can be measured.

Further improvement of this technique would be the introduction of target rotation during argon sputtering (which will reduce the potential sputtering roughness), and the use of an argon gun with higher ion current densities (reduction of the total measuring/sputtering time).
